# Latrodectus envenomation in Ethiopia

**DOI:** 10.1186/s12245-023-00576-z

**Published:** 2024-01-02

**Authors:** Shimelis Korbu, Mosisa Olika, Getu Alemayehu

**Affiliations:** 1 Department of Emergency Medicine and Critical Care, Adama Hospital Medical College, Adama, Ethiopia; 2 Department of Internal Medicine PGY-2 Resident, Adama Hospital Medical College, Adama, Ethiopia

**Keywords:** Black Widow, Spider, Envenomation, Widow

## Abstract

Black widows, one of the few spiders that can sting humans with poison, are members of the spider genus Latrodectus and are well-known for the extraordinary potency of their neurotoxic venom. Latrodectism, a symptom marked by excruciating muscular pain, stomach pain, and diaphoresis after envenomation, is very typical. We described a black widow envenomation case that produced a significant reaction, including diaphoresis and excruciating pain throughout the left thigh that later spread to the lower leg, lower back, belly, and chest. Because of the patient’s description of the spider that bit him and his typical clinical state, it was assumed that Latrodectus envenomation was the cause of his symptoms. The patient received 3 days of observation in the ED while receiving opioid analgesic pain management and muscle relaxant treatment with diazepam. The patient's pain and symptoms were satisfactorily managed, and he was sent home. This case report will help further research be done in the area where it was reported to see if there are cases with similar presentations misdiagnosed as other illnesses. Finally, immediate pain relief is the most critical goal for all patients.

## Introduction

Even though there are almost 40,000 species of spiders known to exist, only a small number of them have been linked to medically relevant envenomations. Black widows, one of the few spiders that can sting humans with poison, are members of the spider genus Latrodectus and are well-known for the extraordinary potency of their neurotoxic venom [1]. The genus, which contains 30 officially identified species, is found all over the world, including in China, Africa, Central Asia, Southern Europe, North and South America, India, and Australia [2]. In Africa, black widow spider populations are not widely distributed. The southern regions of the continent, including nations like South Africa and Namibia, are known to be home to these spiders. The lack of adequate habitats and climates for them to grow in may be the cause of their restricted distribution (https://spidersfaq.com/). Although they are uncommon in occupied homes, black widow spiders are frequently found in garages, trash bins, and outbuildings. When a spider is unintentionally disturbed, bites typically come as a defensive response [3]. Latrodectism, a symptom marked by excruciating muscular pain, stomach pain, and diaphoresis after envenomation, is very typical. Agitation, nausea, hypertension, tachycardia, priapism, and fasciculations are among the more severe envenomations that can occur. This is due to the large release of neurotransmitters, primarily acetylcholine and norepinephrine [4, 5]. Opioid analgesics and sedative-hypnotics may be used to treat Latrodectus envenomations [6]. Patients with severe envenomation whose discomfort is resistant to these treatments may benefit from receiving antivenin [7]. Although historically used as a therapy, calcium gluconate has been demonstrated to be ineffective when compared to benzodiazepines and opioids [6]. Since its initial annual report in 1983–2004, no deaths brought on by Latrodectus envenomation have been reported to the American Association of Poison Control Centers [5]. Latrodectus bites have been linked to fatalities in Spain (2001) [8], Greece (2003) [9], Albania (2006) [10], and Madagascar (1994) [11], according to reports. To the best of our knowledge, fewer cases of Latrodectus spider envenomation have been reported in Africa, but there haven't even been any in Ethiopia.

This report outlines the case of a male patient who presented with a black widow spider bite at Adama Hospital Medical College in 2023.

## Case presentation

This is a 40-year-old male patient who was relatively healthy 24 h ago. Currently, he presented to our emergency department at Adama Hospital Medical College after he sustained a spider sting to the lateral aspect of his left knee from what he described as a “black spider” while he was doing work in his farm yard. The spider was inside the trousers that he put on. When he felt a sudden, severe sting pain on his left later part of the knee, he tried to grab the thing that stung him through his trousers, and he saw a black dead spider with white circular balls underneath his abdomen when he removed his trouser. After 5 min of the incident, the patient started to experience severe pain on the sting site on the lateral aspect of his left knee. The pain progressed to include his left thigh, abdomen, low back, and chest. The patient complained that the pain was so severe that it was difficult for him to walk without support, and he also started to feel lightheaded. The patient was immediately taken to a nearby hospital, where he was given analgesics (tramadol 100 mg IV stat), hydrocortisone 200 mg IV loading, cimetidine 400 mg IV loading, and Tetanus antitoxin 3000 IU stat. Later on, the patient was referred to our emergency department for better management after his pain and condition failed to improve. On his arrival at our emergency department, the patient was having severe pain, cramping, and paresthesias over his left lower extremity (thigh and leg), abdomen, lower back, and chest. He also had two episodes of non-projectile vomiting of ingested matter, diaphoresis, and the inability to extend his left knee. Because of the patient’s description of the spider that bit him and the classic clinical syndrome, his symptoms were believed to be caused by Latrodectus envenomation.

**Fig. 1:**
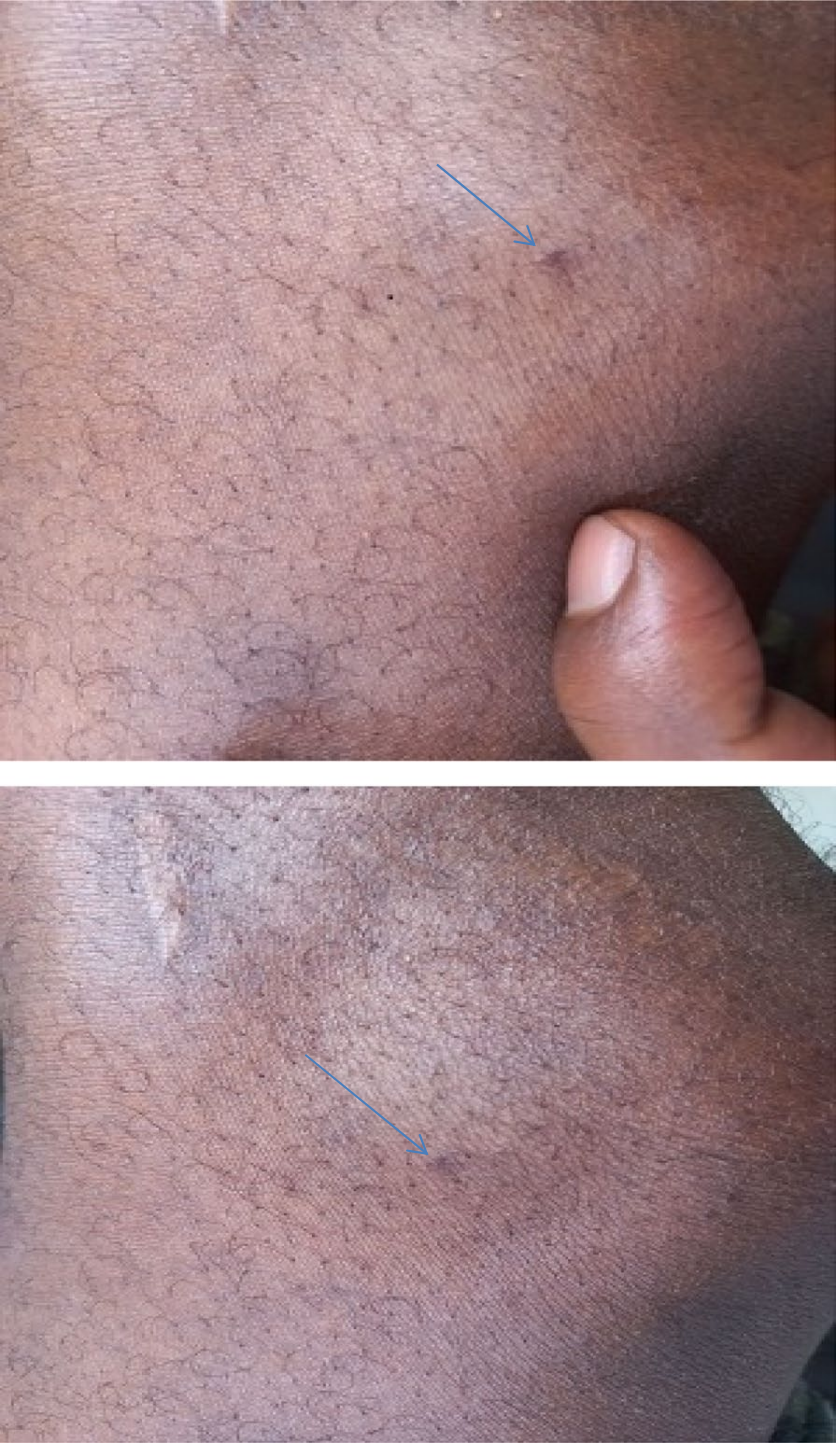
Fang mark to black widow spider bite

His vital signs were the following: at initial presentation to a nearby hospital, blood pressure 150/90; pulse rate 80; respiratory rate 20; and temperature 36 ℃. On his evaluation in our hospital emergency department, his blood pressure was 180/92; pulse rate 56; respiratory rate 20; his temperature was not recorded; his pain score was 10/10; oxygen saturation 92% with atmospheric air; and random blood sugar was 110 mg/dl. He appeared extremely uncomfortable. He was diaphoretic. His lung and cardiac examinations were normal. His abdomen was tight, exhibiting diffuse guarding and engorgement of his penis. There was only a tiny fang mark over the lateral aspect of the left knee (Fig. 1) and tenderness over the lower left extremity. He was initially treated with morphine, which did not provide relief, and later on, diazepam was added, which gave him relief. He was observed in the emergency department for 3 days and then discharged to his home with improved symptoms. A follow-up appointment was scheduled for a week later, but he did not keep it.

**Fig. 2:**
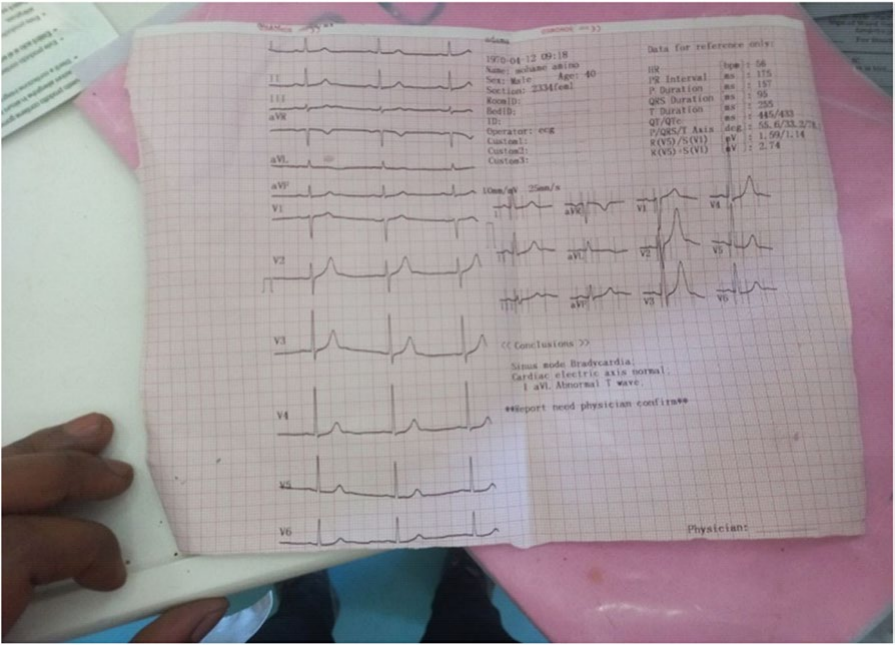
Initial ECG of the patients showing sinus bradycardia with LVH (Note: the calibration of the ECG was not corrected)

His first laboratory results were as follows: CBC was not collected, AST 60.4 μL (normal value 0–40 μL), sodium 129 mmol/L (normal value 136–145), chloride: 91.9 mmol/L (normal value 98–107), and troponin I 0.03 (normal value 0.00–0.1 ng/mL), and the other laboratory results were normal. There is a concentric thickening of the LV wall. The global systolic function of the LV is normal, with EF estimated at 60%. RV systolic function is preserved with a TAPSE of 18 mm. There is mild LV diastolic dysfunction that was detected on echocardiographic examination. The result of the admission electrocardiogram (ECG) was sinus bradycardia (Fig. 2), which was later corrected without management, and left ventricular hypertrophy. Chest radiography was normal. Based on the ECG and echocardiography findings, the patient was considered to have undiagnosed chronic hypertension and put on antihypertensive medication. The investigations were not repeated later on because of the patient’s financial situation.

## Discussion

In cases of latrodectus envenomation, it is crucial that medical professionals identify the symptoms as soon as possible to start providing the best care for patients. The doctor must observe the suspected spider in order to make the proper diagnosis of latrodectus envenomation. Latrodectus bites can be identified with some investigative work if this is not achievable. A characteristic “target” lesion can be seen to help with diagnosis. Additionally, some modest physical examination findings may be beneficial. Asking patients about the beginning of symptoms, how they discovered their bites, and if they observed the spider can help doctors diagnose Latrodectus bites. The bites of Latrodectus are peculiar. The bite site develops a pale center region with surrounding erythema and frequently exhibits evident fang marks [12]. Additionally, there will likely be some redness and swelling where the bite was made.

The diagnosis, in this case, was based mainly on the clinical findings and the patient's account since the spider that needed to be identified was not present. This Latrodectus bite victim displayed the classic signs of a poisonous spider bite. Compared to other insect species, these symptoms were more severe. Our patient experienced profound diaphoresis, generalized back pain, hypertension, and other symptoms seen in people with significant Latrodectus envenomations [6]. Despite the fact that some patients will experience severe cardiac manifestations [9], we think that the echocardiographic and electrocardiographic findings seen in this patient were caused by untreated chronic hypertension rather than envenomation.

The cleansing of the bite site is reasonable. The best way to treat latrodectism is still up for debate. To alleviate pain, the administration of opioids is appropriate, and benzodiazepines may also be taken into consideration. Admission can be required for severe envenomations in order to get effective pain relief [6, 13]. Additionally, calcium therapy used to be thought of as a treatment for black widow envenomation. It was anticipated that calcium would reduce neurotransmitter release by stabilizing the permeability of neuronal membranes. Although this effect was proven to exist in vitro and was mentioned in a few early clinical studies, further experience has not supported its efficacy. As a result, calcium treatment is no longer widely used in the field of medical toxicology. Opioid analgesics and black widow spider venom are the only treatments with a track record of success [6, 13, 14]. Since there is no approved antivenom for such circumstances, our patient was initially treated with opiate analgesics before benzodiazepam was introduced to help him manage his symptoms.

## Conclusion

In the ED, spider bites are not a frequent concern. Ethiopia has not yet experienced any reports of black widow envenomation. In order to find out if there are more instances with a similar presentation that were incorrectly identified as having another condition, further research in the area of the case where it was recorded will benefit from this case report.

In order to treat Latrodectus bite victims right away, healthcare professionals must be able to detect the signs and symptoms of the disease. Supportive care is the main treatment strategy for mild-to-moderate Latrodectus envenomations. Hospitalization and potentially the administration of antivenin should be saved for patients with severe systemic symptoms or insufficient pain management, which is not possible in our system. Finally, prompt pain alleviation is the most crucial objective for all patients.

## Data Availability

Data and materials are available and can be shared by the corresponding author.
